# The Landscape of Breast Cancer Molecular and Histologic Subtypes in Canada

**DOI:** 10.3390/curroncol31090411

**Published:** 2024-09-17

**Authors:** Anna N. Wilkinson, Larry F. Ellison, Sharon F. McGee, Jean-Michel Billette, Jean M. Seely

**Affiliations:** 1 Department of Family Medicine, University of Ottawa, Ottawa, ON K1H 8L6, Canada; 2 Centre for Population Health Data at Statistics Canada, Government of Canada, Ottawa, ON K1A 0T6, Canada; larry.ellison@statcan.gc.ca (L.F.E.);; 3 The Ottawa Hospital Cancer Centre, University of Ottawa, Ottawa, ON K1H 8L6, Canada; shmcgee@toh.ca; 4 Department of Radiology, The Ottawa Hospital Research Institute, University of Ottawa, Ottawa, ON K1H 8L6, Canada; jeseely@toh.ca

**Keywords:** breast cancer, molecular subtype, histology, net survival, outcomes, stage, registries

## Abstract

**Purpose:** To characterize the histologic and molecular subtype distribution of, and survival from, breast cancer (BC) among Canadian women overall, and by stage and age at diagnosis. **Methods:** Invasive BC cases from the Canadian Cancer Registry for women aged 15–99 years between 2012 and 2017 in Canada, excluding Quebec, were examined using pre-existing mortality linkages. Stage at diagnosis, molecular, and histologic subtypes, and 5-year net survival (NS) by age, subtype, and stage were determined. **Results:** 107,271 women with BC were included. Luminal A was the most common subtype, present in increasing proportions as women aged, up to a maximum of 55% of cases in 70–74. Ductal and luminal A were most likely to be diagnosed at stage I, while HER2+ had the highest proportion of diagnosis at stage III; triple negative (TN) and unknown had the highest proportion of stage IV. For all stages combined, luminal A had a five-year NS of 98%, while TN was 74%. NS for stage I BC was 99–100% for all subtypes, excepting TN, which was 96%. Survival decreased with advancing stage, most markedly for TN, for which stage III was 47% and stage IV 7%. Survival by equivalent stage and subtype was comparable across age groups but declined in older age categories. **Conclusions:** The varying natural histories of BC subtypes and histologies can inform prognoses, health system economics, and screening practices. The NS of 96% or greater for stage I, regardless of subtype, highlights the importance of early detection for all subtypes of BC, especially in aggressive subtypes.

## 1. Introduction

Breast cancer (BC) is not a single biological entity; rather, it is a collection of diseases characterized by discrete histological and molecular subtypes [1]. Ductal and lobular carcinomas are the most common invasive BC histologies. In the absence of definitive gene expression profiling, molecular subtype can be assigned using clinicopathologic elements such as her2/neu (HER2), expression, tumour grade, and the expression of hormone receptor status including estrogen (ER) and progesterone receptors (PR) [2,3]. Recognized molecular subtypes of BC are luminal A (ER+, PR+, HER2−), luminal B (ER+, PR+/−, HER2+/−), HER2 (ER-, HER2+), and triple negative (TN) (ER-, HER2-) [1,4]. A further division into five subgroups reclassifies some luminal A and B cases into luminal B-like, accounting for the importance of variations in PR expression and Ki-67 level, or its proxy, grade [5].

Luminal A BC typically has a better prognosis, and recurrences may occur many years (>10) after treatment [4]. Luminal B and B-like cancers are generally higher grade with increased proliferation and have a poor prognosis as they are relatively resistant to chemotherapy and can have suboptimal response to endocrine therapy [6]. Recurrence of HER2+ and TN cancers is common and typically seen within 3–5 years after treatment [7]. HER2+ cancers can be markedly sensitive to chemotherapy and HER2-targeted therapy, and, in this case, can have a better prognosis [8]. TN BC grows rapidly, often presenting with larger tumours or interval cancers [9]. Although TN reportedly constitutes 11–20% of BC, it accounts for up to 28% of locally advanced disease [10]. The subtypes have differing preferential metastatic sites, with luminal A commonly metastasizing to bones, HER2+ cancers to the brain, and TN to visceral organs [9,11]. Survival has been shown to be highest for luminal A BC, and is initially lowest for TN, although over a period longer than ten years, luminal B survival may decline beyond that of TN [8,12].

Increasing the granularity of our knowledge of the molecular makeup of BC in Canada is critical as these characteristics allow for precision therapies and are predictive of treatment response, recurrence rates, site of metastases, and prognosis [6,8]. Given the vastly different costs for the treatment of varying BC molecular subtypes [13], a clear understanding of the distribution of these subtypes in our population is important to inform health system economics. However, pan-Canadian data on BC molecular subtypes have only been collected in more recent years by the Canadian Cancer Registry (CCR) and have yet to be characterized in a published report.

This study used HER2, ER, and PR status in association with grade to partition BC cases within the CCR into molecular subtypes. By subdividing BC cases into this level of detail, the molecular and histological details of BC in Canada could be investigated with respect to incidence, age and stage at diagnosis, and net survival (NS).

## 2. Methods

The CCR is a population-based database comprising data annually collected and reported to Statistics Canada by each provincial and territorial cancer registry [14]. Survival and frequency distribution analyses were undertaken using a pre-existing analytic file created by linking cases from the CCR file released 29 January 2020, to mortality information complete through 31 December 2017. More information about this file can be found elsewhere [15]. Based on the International Classification of Diseases for Oncology, Third Edition [16], only primary invasive BC cases (site code C50) were included [17,18]. Ductal carcinoma in situ (DCIS) cases were not included. This study was a secondary analysis of nationally deidentified data collected by Statistics Canada, and, as such, ethics approval was not required.

All analyses were based on cases diagnosed from 2012 to 2017, the period for which the variables necessary to determine molecular subtype were consistently reported to the CCR. All new primary BC cases diagnosed in women aged 15 to 99 years were initially included. Cases from the province of Quebec were not available for this period on this file. Cases with an undefined survival time (e.g., cases for which the diagnosis had been established through autopsy or death certificate only) were necessarily excluded. Only the first BC case diagnosed per person on the file was considered [19,20]. Of the remaining 110,407 cases, 0.25% were excluded based on stage data, because they were either considered out of scope for survival analysis (i.e., noninvasive stage 0 cases staged as occult) or coded as “not applicable” (i.e., unstageable), and 2.59% were excluded because the stage variable was coded as a missing value (i.e., unstaged cases). A total of 107,271 BC cases were included in the analyses.

The 7th edition of the American Joint Committee on Cancer’s (AJCC) Cancer Staging Manual was used to determine the stage at diagnosis, as specified by the Collaborative Stage Data Collection System. Based on the tumour, node, and metastasis staging system, cancers are usually assigned an overall stage grouping categorized as either 0, I, II, III, IV, or unknown [21]. The 7th edition of the AJCC was used as the cancers included were reported in 2017 and earlier. The unknown stage category is restricted to cases where staging was attempted but the collected information was insufficient to determine a specific stage. In contrast, missing stage refers to cases for which staging was not attempted (i.e., unstaged).

BC cases with a histology code of 8500 (infiltrating duct carcinoma) were classified as ductal, while those with a code of 8520 (lobular carcinoma) were classified as lobular. Cases with any other histology code were combined to form the “other” category. In the absence of immunohistochemical information on Ki-67 levels, BC cases were classified into five molecular subtypes according to the following scheme: luminal A: (ER positive and PR positive, HER2 negative and low/intermediate grade on the Nottingham or Bloom-Richardson grading scale); luminal B (ER positive and PR positive, HER2 negative and high grade, or ER positive, PR negative, HER2 negative, and any grade); luminal B-like (ER positive and PR positive or negative, HER2 positive, any grade); HER2-enriched (ER negative, PR negative, HER2 positive); and triple negative (ER negative, PR negative, HER2 negative). Additionally, a residual category was created to retain BC cases that were impossible to classify due to either missing or unrecorded hormone receptor information.

BC NS estimates were derived using an algorithm [22] that has been augmented by Ron Dewar of the Nova Scotia Health Cancer Care Program to include the Pohar Perme estimator of NS using the hazard transformation approach (Dewar R, 2020, email communication, 22 June) [23]. The complete approach, in which the follow-up from diagnosis to either death or end of study period of all eligible cases is included in the analysis, was used [24]. Expected survival probabilities, necessary for the calculation of NS in a relative survival framework, were mostly obtained from sex-specific complete annual provincial population life tables [25]. Further detail on the calculation of expected survival is provided elsewhere [26]. NS estimates cancer survival in the hypothetical case where other competing causes of death are removed.

Two-sided statistical tests of the null hypothesis that differences in proportions or NS estimates were zero, with a significance level of 0.05, were performed on ad hoc basis to support the description and interpretation of results.

## 3. Results

### 3.1. Distribution Analysis

**Figure 1:**
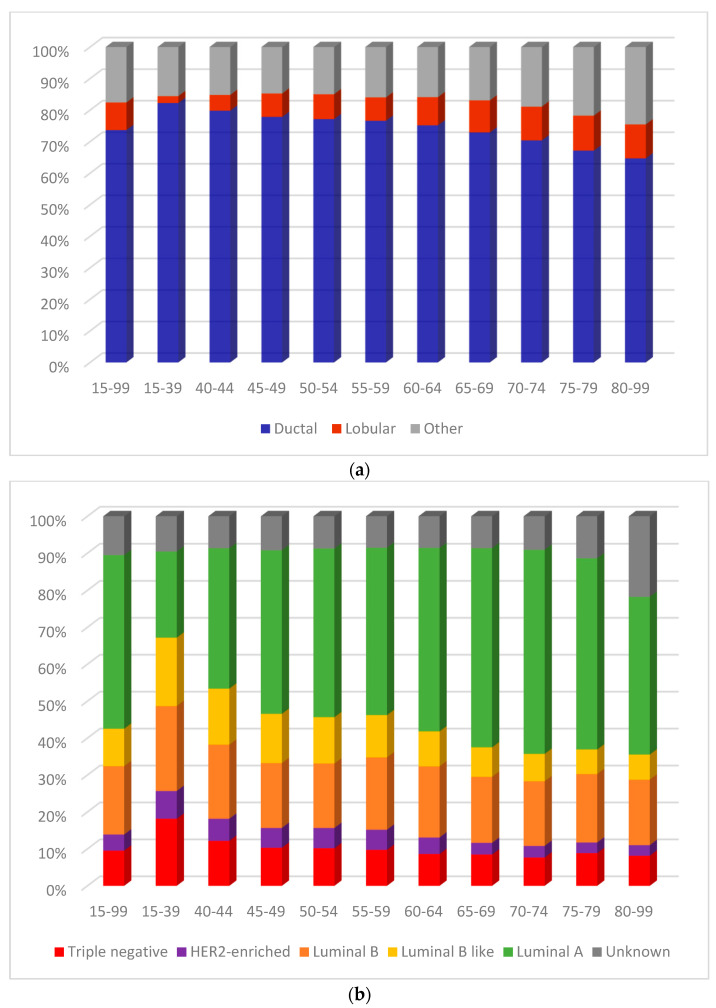
(**a**) Female breast cancer histologic subtype distribution by age group, ages 15 to 99 years, Canada excluding Quebec, 2012 to 2017 diagnosis period. (**b**) Female breast cancer molecular subtype distribution by age group, ages 15 to 99 years, Canada excluding Quebec, 2012 to 2017 diagnosis period. Notes: Quebec is excluded because cases diagnosed in that province from 2011 onward had not been submitted to the Canadian Cancer Registry at the time that the source file was created. Source: Statistics Canada, Canadian Cancer Registry death linked file (1992 to 2017).

The majority of the 107,271 BC cases included in the study were ductal (73.7%), 8.7% were lobular, and 17.6% were other histologies (Figure 1a, Table 1a, Appendix A Table A1). The proportion of cases that were ductal monotonically decreased with advancing age from 82.2% among women 15–39 to 64.7% among women 80–99. In contrast, the proportion of cases identified as lobular generally increased with age from 2.2% (15–39) to 10.8% (80–99). Across all ages, luminal A was the most diagnosed BC molecular subtype (47.0%), followed by luminal B (18.5%) (Figure 1b, Table 1b). The least diagnosed subtype was HER2+ (4.4%). The remaining 30.1% was similarly divided between luminal B-like (10.1%), TN (9.5%), and cases for which the molecular subtype was unknown (10.5%). The molecular subtype distribution varied considerably with age. For example, the proportion of cases diagnosed as luminal A was generally greater at older ages increasing from 23.3% of cases among women 15–39 to a peak of 55.2% among women 70–74. For luminal B-like, HER2+ and TN, proportions were highest in the 15–39 group (18.5%, 7.5%, and 18.2%, respectively), and declined with increasing age. The proportion diagnosed with the luminal B subtype remained stable, at around 18%, in most age groups. Similarly, the proportion of cases with unknown molecular subtype was stable at about 9% but increased dramatically to 21.7% among women aged 80–99.

**Figure 2:**
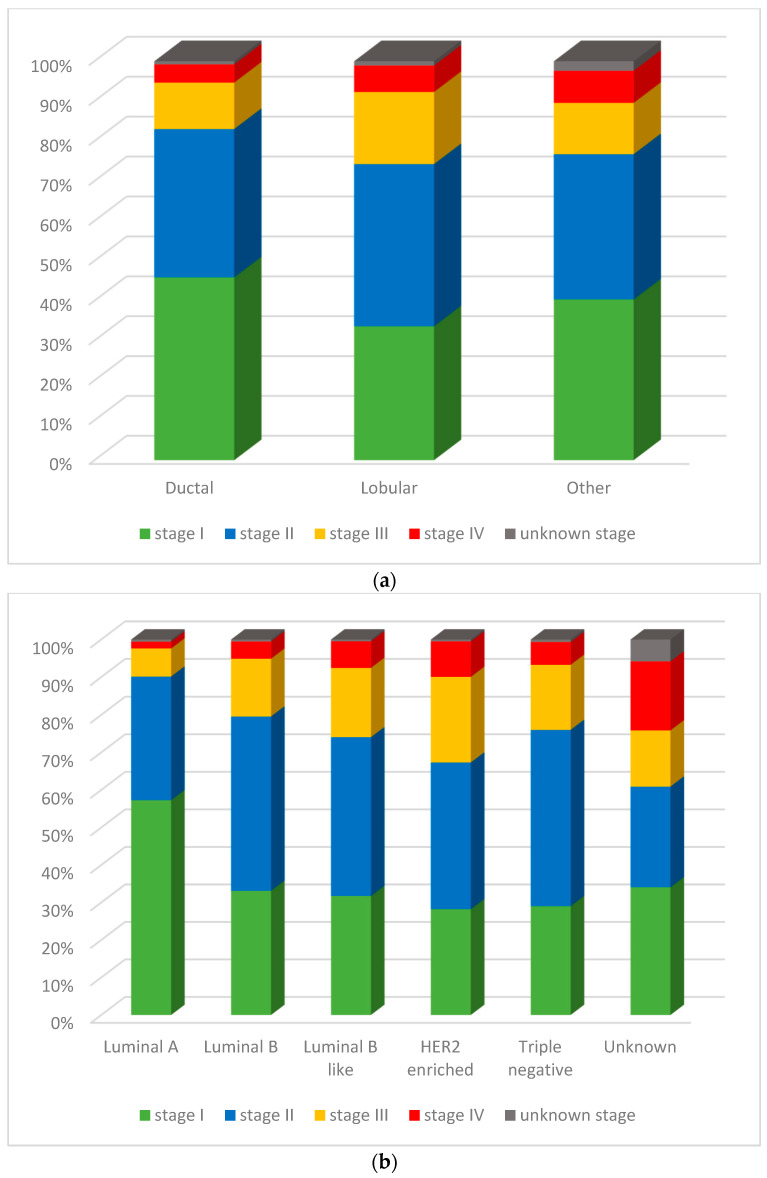
(**a**) Female breast cancer stage at diagnosis distribution by histologic subtype, ages 15 to 99 years, Canada excluding Quebec, 2012 to 2017 diagnosis period. (**b**) Female breast cancer stage at diagnosis distribution by molecular subtype, ages 15 to 99 years, Canada excluding Quebec, 2012 to 2017 diagnosis period. Notes: Quebec is excluded because cases diagnosed in that province from 2011 onward had not been submitted to the Canadian Cancer Registry at the time that the source file was created. Source: Statistics Canada, Canadian Cancer Registry death linked file (1992 to 2017).

Ductal BC was more likely to be diagnosed at stage I than lobular or all other histologies combined (Figure 2a). Lobular BC had a higher proportion (65.3%) of more advanced stage (i.e., II, III, and IV) cases at diagnosis than ductal (53.3%). In terms of molecular subtypes, luminal A was associated with the highest proportion of stage I BC at diagnosis (57.3%), and the lowest proportions of both stage III (7.5%) and IV (1.8%) (Figure 2b). The lowest proportions of stage I BC were observed for HER2+ (28.2%) and TN (29.1%). TN (46.9%) and luminal B (46.4%) were the molecular subtypes with the highest proportion of cases diagnosed at stage II, and the lowest proportions of stage IV cases (6.1% and 4.6%, respectively) after luminal A (1.8%). HER2+ had the highest proportion at stage III (22.7%) and, apart from the unknown subtype, the highest proportion at stage IV (9.4%).

### 3.2. Net Survival Analysis

**Figure 3:**
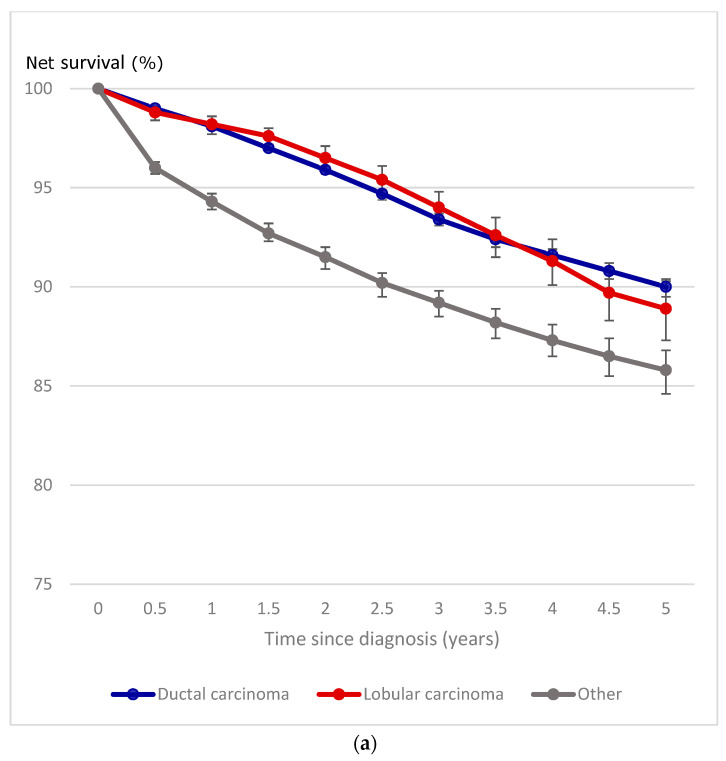

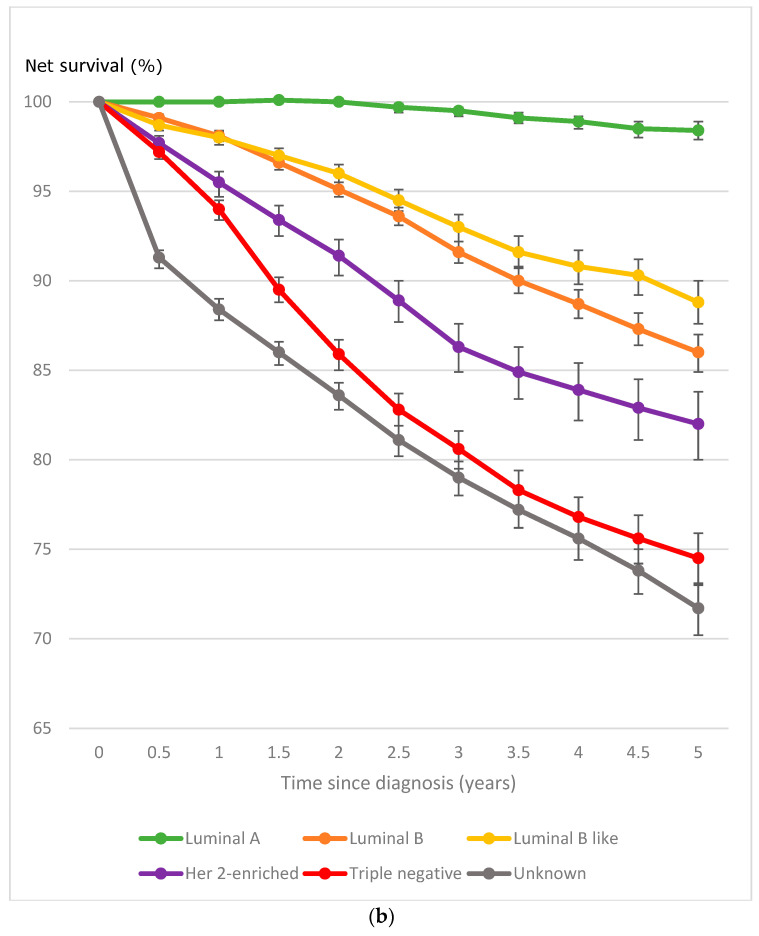
(**a**) Five-year cumulative female breast cancer net survival, by histologic subtype, ages 15 to 99 years, Canada excluding Quebec, 2012 to 2017 diagnosis period. (**b**) Five-year cumulative female breast cancer net survival, by molecular subtype, ages 15 to 99 years, Canada excluding Quebec, 2012 to 2017 diagnosis period. Notes: Quebec is excluded because cases diagnosed in that province from 2011 onward had not been submitted to the Canadian Cancer Registry. Follow-up of cases is available to the end of 2017. Overlaid vertical bars denote 95% confidence intervals. Confidence intervals are undefined for unrounded point estimates of 100% or greater. Sources: Statistics Canada, Canadian Cancer Registry death linked file (1992 to 2017) and life tables.

Ductal and lobular BC had similar 5-year NS of 90% and 89%, respectively (Figure 3a). However, survival for both types was higher than among other histologic subtypes cases combined (86%). BC NS among women differed significantly by molecular subtype (Figure 3b). Five-year NS was highest among luminal A cases (98%), the only subtype with an NS exceeding 90%. The next highest estimates were observed among women diagnosed with luminal B-like (89%), luminal B (86%), and HER2+ (82%) subtypes. Excluding cases with an unknown molecular subtype that collectively had a 5-year NS of 72%, survival was lowest among TN cases (74%).

Five-year, stage-specific NS was similar among ductal and lobular BC, although survival among lobular cases was higher for stage II (96% vs. 92%) and lower for stage III (73% vs. 78%) (Table 2). Five-year NS at stage I ranged between 99% and 100% for all female BC molecular subtypes except for TN, which had an NS of 96%. Within each subtype, declines in NS were observed with increasing stage at diagnosis such that stage IV NS ranged from 7% (TN) to 38% (luminal A). The magnitude of these declines was more pronounced at later stages—absolute declines in survival estimates from stage I to stage II ranged from 3 (luminal A) to 15 (TN) percentage points, while between stage III and IV they ranged from 40 (TN) to 51 (luminal A and B) percentage points. At each given stage, 5-year NS point estimates were highest among luminal A cases followed by luminal B-like cases; excepting unknown subtype in some instances, they were lowest for TN cases. NS by stage for luminal B and HER2+ cases was very similar.

Five-year NS among women diagnosed with luminal A BC was approximately 100% for all age groups except 15–39 (93%). NS was also lower in the 15–39-year group versus the 40–40 group among luminal B cases (83% versus 88%). Among luminal B-like cases, NS was approximately 90% up to and including the 60–69 age group, then decreased, ultimately to 78% in the 80–99 age group. NS percentages among luminal B and HER2+ cases were consistently in the 80s apart from the 80–99 age group in which they were lower. Similarly, NS was also lowest in the last age group among TN cases, otherwise percentage estimates were in the 70s. Among women with ductal BC, 5-year NS ranged from 89% to 92% for cases diagnosed between the age groups of 40–49 and 70–79 (Table 3). The corresponding range among lobular BC cases was 87% to 93%. Survival was lowest in women 80 or older at diagnosis (82% ductal, 84% lobular), and in women younger than 40 (84% ductal, 87% lobular). However, these between-age-group differences were not statistically significant among lobular cases.

## 4. Discussion

This study highlights that BC is not a single biological entity. Molecular and histological subtypes are present in varying proportions in different age groups and have unique characteristics in terms of stage at diagnosis and survival outcomes. The five-year prognosis for all stage I BC, regardless of subtype, is excellent. The NS for all subtypes and stages was lower in women at the younger and older extremes of age. Most BCs in Canada are luminal A, especially in older women. Luminal A cancers have very positive stage distributions and outcomes. TN and HER2+ have the poorest prognosis with the lowest NS, aside from unknown cases. These two subtypes are preferentially diagnosed among younger women, and when the disease has progressed beyond stage I.

The results from this study are similar to what has been reported in the US, with almost identical proportions of ductal and lobular BC and similar prognoses [27,28]. Luminal A has previously also been noted to be the most common subtype and to have the highest survival [11,29,30,31]. The proportion of TN cases in this study (9.5%) aligned with results previously reported in Ontario Canada (8.6%) and the US (11.3%), while the HER2+ proportion (4.4%) was also similar to the 4.0% seen previously in Ontario, and the 4.8–6.4% in the US [11,29,30,31]. Breast cancer specific survival from this study corroborates previous subtype specific estimates noted in the US [32].

The high NS regardless of subtype for stage I BC suggests that earlier detection of BC translates into improved clinical outcomes. Earlier detection may be contributing to the highest NS which is seen in the 50–69-year-old age groups, women for whom organized screening programs exist [33]. The use of NS, which isolates survival to the impact of cancer alone, shows that survival due to BC is fairly stable from ages 50–79, and only starts to appreciably decline after age 80, excepting NS for luminal A, which does not decrease. This may be because women older than 80 are no longer included in screening programs, or because older women tolerate or accept endocrine therapy but not chemotherapy. Cases with unknown stage or molecular subtype were more common in women older than 80. Unknown subtype cases also had poorer overall NS in the first couple of years after diagnosis than the aggressive TN subtype. It has previously been observed that NS for cancers with unknown stage is intermediate to stage III and IV [15], and, given the predominance in older women, may represent the presentation of advanced disease in individuals who are too frail or have too many comorbidities to allow for fulsome pathological or staging investigations.

The differing natural history of subtypes and histologies are highlighted in this study. TN was more commonly diagnosed at stages III and IV compared to luminal A BC (23% compared to 9%), and lobular BC was also more commonly diagnosed at an advanced stage than ductal BC. NS in TN BC was 75%, compared to 97% for luminal A, with NS for stage III TN BC only 47%, compared to 89% for stage III luminal A BC. Later stage diagnosis may be partially explained by differing tumour volume doubling times [34], and differing ability of imaging to detect tumour types. The infiltrative growth pattern of lobular BC hampers detection with mammography, while TN BC can simulate benign tumors, and the presence of calcifications and/or spiculations in luminal A tumors results in easier detection [35,36,37]. However, screen-detected TN are documented to be diagnosed at an earlier stage than clinically detected TN BC, and have a 5-year overall survival of 92.8% compared to 81.5% for non-screen-detected TN BC [38]. When quantified, screening has the greatest contribution to mortality reduction for TN, at 40%, compared to 24%, 19%, and 24.5% for HER2+, luminal B, and luminal A, respectively [39]. TN is known to be more common in black and Hispanic women and is proportionally greatest in women younger than 50 [8,38,40]. Populations with higher proportions of aggressive subtypes and resultant increased rates of associated later stage cancers with poorer prognoses may gain the most benefit from early detection with screening and have fewer concerns around overdiagnosis and lead time bias [41].

This knowledge of the incidence of molecular subtypes by age and stage distribution is critical information to inform health system planning. The costs of BC therapy vary widely based on stage and subtype (e.g., stage I luminal A CAD 28,201; stage III TN CAD 110,798) [13]. Thus, the national picture by subtype and stage presented herein may be used to more accurately reflect the true cost of treating BC and improve economic forecasting for BC cancer therapy costs. Additionally, knowledge of the predominate subtypes present at each age, coupled with the tumour doubling velocity for each subtype, could be used to help guide screening recommendations and intervals.

Limitations for our study include the lack of available information on Ki67 for BC cases, the presence of which could help to delineate luminal B and luminal B-like cases more accurately. The absence of race and ethnicity information for BC cases precluded investigation of the relationship of molecular subtype with race. The 7th edition of the AJCC was in use from 2012 to 2017, so translation of these findings to cases staged using the current 8th edition of the AJCC should be undertaken with caution. The ongoing evolution of BC therapies, such as the use of immunotherapy in TN BC, may mean that NS has improved since the study period [42]. Cases with an unknown molecular subtype were not assigned one through imputation, which could have led to an overestimation of NS as such cases may be associated with worse prognostic characteristics [42]. The method of BC detection (e.g., screen-detected or symptomatic), which could have been valuable to correlate with molecular subtype, was unavailable. Finally, a five-year period for NS is likely not capturing the full story of survival for BC molecular subtypes, given that recurrence for TN and HER2+ BCs commonly occurs three to five years after diagnosis, and luminal A much longer than this.

This study analyzed CCR cases in terms of BC histology and molecular subtype to generate critical information on incidence by age, stage at diagnosis, and survival outcomes for Canadian women. Importantly, BC NS is highest for all histologies and subtypes when diagnosed at stage I, illustrating that early-stage detection is beneficial regardless of BC histology or subtype. The information from this study can be used to better prognosticate for patients at diagnosis based on their molecular subtype, to inform health system economics, and to appreciate differing proportions of subtypes throughout age trajectories, which can inform screening practices.

## Figures and Tables

**Table 1:** (**a**) Age group and stage at diagnosis distribution of female breast cancer cases by histologic subtype, ages 15 to 99 years, Canada excluding Quebec, 2012 to 2017 diagnosis period. (**b**) Age group and stage at diagnosis distribution of female breast cancer cases by molecular subtype, ages 15 to 99 years, Canada excluding Quebec, 2012 to 2017 diagnosis period.

**(a)**							
**Age Group/Stage**	**Histologic Subtype**						
	**Infiltrating Ductal** **Carcinoma**			**Lobular** **Carcinoma**		**Other**	
	**n = 79,039**			**n = 9369**		**n = 18,863**	
**Age group**							
15–39	5.0%			1.1%		3.9%	
40–49	14.9%			10.4%		11.8%	
50–59	24.7%			20.7%		20.8%	
60–69	27.3%			29.7%		25.4%	
70–79	17.8%			23.6%		21.6%	
80–99	10.3%			14.5%		16.4%	
**Stage at diagnosis**							
I	45.9%			33.6%		40.4%	
II	37.1%			40.6%		36.4%	
III	11.6%			18.1%		12.8%	
IV	4.5%			6.6%		8.0%	
Unknown	0.8%			1.1%		2.4%	
**(b)**							
**Age group/stage**	**Molecular subtype**						
	**All subtypes**	**Luminal** **A**	**Luminal** **B**	**Luminal** **B like**	**HER-2** **enriched**	**Triple** **negative**	**Unknown**
	**n = 107,271**	**n = 50,394**	**n = 19,859**	**n = 10,854**	**n = 4684**	**n = 10,220**	**n = 11,260**
**Age group**							
15–39	4.5%	2.2%	5.5%	8.1%	7.7%	8.5%	4.0%
40–49	14.0%	12.5%	14.0%	19.3%	18.0%	16.2%	11.9%
50–59	23.7%	22.9%	23.7%	28.0%	29.7%	24.7%	19.4%
60–69	27.2%	30.0%	27.2%	23.3%	23.8%	24.4%	22.2%
70–79	19.0%	21.8%	18.3%	13.3%	13.2%	16.2%	18.1%
80–99	11.7%	10.7%	11.2%	7.9%	7.7%	10.0%	24.3%
**Stage at diagnosis**							
I	43.8%	57.3%	33.1%	31.7%	28.2%	29.1%	34.1%
II	37.3%	32.8%	46.4%	42.3%	39.1%	46.9%	26.8%
III	12.4%	7.5%	15.3%	18.4%	22.7%	17.3%	15.0%
IV	5.3%	1.8%	4.6%	7.1%	9.4%	6.0%	18.3%
Unknown	1.1%	0.6%	0.5%	0.5%	0.5%	0.7%	5.8%

Notes: Quebec is excluded because cases diagnosed in that province from 2011 onward had not been submitted to the Canadian Cancer Registry at the time that the source file was created. Column totals by age group and by stage at diagnosis may not sum to 100.0% due to rounding. Source: Statistics Canada, Canadian Cancer Registry death linked file (1992 to 2017).

**Table 2:** Five-year stage-specific female breast cancer net survival estimates and 95% confidence intervals by molecular and histologic subtype, ages 15 to 99 years, Canada excluding Quebec, 2012 to 2017 diagnosis period.

Stage at Diagnosis	Molecular Subtype							Histologic Subtype		
	Luminal A	Luminal B	Luminal B Like	HER-2 Enriched	Triple Negative	Unknown		Infiltrating Ductal Carcinoma	Lobular Carcinoma	Other
I	102 (..)	98 (96–99)	100 (..)	99 (92–100)	96 (94–97)	96 (93–98)		100 (85–100)	101 (..)	101 (..)
II	99 (97–99)	90 (88–91)	93 (91–94)	90 (87–92)	81 (79–83)	78 (74–82)		92 (91–93)	96 (93–98)	91 (88–92)
III	89 (87–91)	70 (67–73)	81 (78–84)	71 (67–75)	47 (43–51)	65 (61–70)		73 (72–75)	78 (74–82)	73 (69–75)
IV	38 (32–44)	19 (15–24)	34 (29–40)	27 (20–35)	7 (4–10)	19 (16–22)		26 (23–28)	23 (18–29)	18 (14–22)
Unknown	70 (56–81)	44 (25–62)	…	…	43 (24–60)	51 (43–58)		63 (54–71)	…	47 (39–56)

Notes: Quebec is excluded because cases diagnosed in that province from 2011 onward had not been submitted to the Canadian Cancer Registry at the time that the source file was created. Estimates are expressed as percentages. Caution should be used in interpreting estimates associated with an unrounded standard error greater than 0.05 and smaller than or equal to 0.10. Such estimates are presented with lightly shaded background. Estimates associated with a standard error greater than 0.10 were considered too unreliable to be published. Confidence intervals are undefined for unrounded point estimates of 100% or greater. Follow-up of cases is available to the end of 2017. Sources: Statistics Canada, Canadian Cancer Registry death linked file (1992 to 2017) and life tables. .. not available; … too unreliable to be published.

**Table 3:** Five-year age-specific female breast cancer net survival estimates and 95% confidence intervals by molecular and histologic subtype, ages 15 to 99 years, Canada excluding Quebec, 2012 to 2017 diagnosis period.

Age at Diagnosis	Molecular Subtype							Histologic Subtype		
	Luminal A	Luminal B	Luminal B Like	HER-2 Enriched	Triple Negative	Unknown		Infiltrating Duct Carcinoma	Lobular Carcinoma	Other
15–99	98 (98–99)	86 (85–87)	89 (88–90)	82 (80–84)	74 (73–76)	72 (70–73)		90 (89–90)	89 (87–90)	86 (85–87)
15–39	93 (90–96)	83 (79–87)	89 (85–93)	85 (78–89)	73 (68–77)	83 (78–87)		84 (83–86)	87 (73–94)	87 (83–90)
40–49	98 (97–99)	88 (86–90)	91 (89–93)	84 (80–87)	76 (73–79)	86 (83–88)		91 (90–92)	93 (90–95)	89 (87–91)
50–59	98 (97–98)	87 (85–88)	92 (90–94)	88 (85–90)	79 (77–81)	82 (80–84)		92 (91–92)	92 (89–94)	88 (87–90)
60–69	98 (97–98)	89 (87–90)	91 (88–93)	82 (78–85)	78 (76–81)	76 (74–79)		92 (91–93)	90 (87–91)	89 (87–90)
70–79	98 (97–99)	85(82–87)	82 (77–85)	81 (75–86)	71 (66–74)	67 (64–70)		89 (88–90)	87 (83–89)	87 (85–89)
80–99	103 (..)	78 (72–83)	78 (68–85)	54 (42–65)	59 (51–66)	52 (47–56)		82 (79–85)	84 (75–90)	73 (68–78)

Notes: Quebec is excluded because cases diagnosed in that province from 2011 onward had not been submitted to the Canadian Cancer Registry at the time that the source file was created. Estimates are expressed as percentages. Caution should be used in interpreting estimates associated with an unrounded standard error greater than 0.05 and smaller than or equal to 0.10. Such estimates are presented with lightly shaded background. Confidence intervals are undefined for unrounded point estimates of 100% or greater. Follow-up of cases is available to the end of 2017. Sources: Statistics Canada, Canadian Cancer Registry death linked file (1992 to 2017) and life tables. .. not available.

## Data Availability

Data are accessible in the Canadian Cancer Registry.

## Appendix A

**Table A1:** Distribution of the most common subtypes within the “other” histologic subtype category, as a percentage of the overall total number of female breast cancer cases analyzed (n = 107,271), Canada excluding Quebec, 2012 to 2017 diagnosis period.

Histologic Subtype	Cases	%
Infiltrating duct and lobular carcinoma	4449	4.1%
Infiltrating duct mixed with other types of carcinoma	3619	3.4%
Carcinoma, not otherwise specified	2079	1.9%
Mucinous adenocarcinoma	2000	1.9%
Intraductal micropapillary carcinoma	926	0.9%
Metaplastic carcinoma, not otherwise specified	684	0.6%
Tubular adenocarcinoma	564	0.5%
Adenocarcinoma, not otherwise specified	564	0.5%
Apocrine adenocarcinoma	545	0.5%
Neoplasm, malignant	527	0.5%
Intraductal papillary adenocarcinoma with invasion	478	0.4%

Note: Quebec is excluded because cases diagnosed in that province from 2011 onward had not been submitted to the Canadian Cancer Registry at the time that the source file was created. Source: Statistics Canada, Canadian Cancer Registry death linked file (1992 to 2017).
